# CHL1 Is Expressed and Functions as a Malignancy Promoter in Glioma Cells

**DOI:** 10.3389/fnmol.2017.00324

**Published:** 2017-10-17

**Authors:** Zhai Yang, Qing Xie, Cheng-Liang Hu, Qiong Jiang, Hui-Fan Shen, Melitta Schachner, Wei-Jiang Zhao

**Affiliations:** ^1^Center for Neuroscience, Shantou University Medical College, Shantou, China; ^2^Keck Center for Collaborative Neuroscience and Department of Cell Biology and Neuroscience, Rutgers University, Piscataway, NJ, United States

**Keywords:** glioma, glioma cell lines, close homolog of L1 cell adhesion molecule (CHL1), siRNA, malignancy

## Abstract

The cell adhesion molecule with homology to L1CAM (close homolog of L1) (CHL1) is a member of the cell adhesion molecule L1 (L1CAM) gene family. Although CHL1 expression and function have been reported in several tumors, the roles of CHL1 in the development of glioma remain unclear. In the present study, we investigated the effects of CHL1 on proliferation indexes and activation of Akt1 and Erk signaling by siRNA in U-87 MG human glioblastoma and human U251 and SHG-44 glioma cells. We found that siRNA targeting CHL1 significantly down-regulated the expression of CHL1 mRNA and protein accompanied by reduced cell proliferation and transmigration invasion in all three cell lines. Down-regulating CHL1 expression also reduced cell survival, as measured by the Bax/Bcl-2 ratio, and increased activation of caspase-3. In subcutaneous U-87 MG cell xenograft tumors in nude mice, intratumoral administration of siRNA targeting CHL1 treatment significantly down-regulated CHL1 expression *in vivo*, accompanied by increased levels of activated caspase-3. Our combined results confirmed for the first time that in contrast to findings about CHL1 in most other cancer types, CHL1 functions in promoting cell proliferation, metastasis and migration in human glioma cells both *in vitro* and *in vivo*. These results indicate that CHL1 is a therapeutic target in the clinical management of glioma/glioblastoma.

## Introduction

Gliomas are a set of highly invasive glial cell-derived tumors that originate in the central nervous system, and they account for 40% to 50% of all intracranial tumors (Gabriel et al., 2014). Despite advancements in surgery, chemotherapy and radiation oncology technology, the average survival time is 9.7 months. In addition, patients with lowly differentiated astroglioma and glioblastoma exhibit high recurrence, high mortality and low cure rates, and the 5-year survival rate remains less than 5% (Sathornsumetee et al., 2007; Nakazato, 2008; Jemal et al., 2010). All these clinical data suggest that the development of glioma is a multifactor-based process in which a series of molecules are involved.

We previously reported that cell adhesion molecule L1 (L1CAM) is involved in regulating tumor progression and invasion under the modulation of Neuregulin-1 (Nrg1), suggesting the roles of L1 family members in glioma development (Zhao and Schachner, 2013). The close homolog of cell adhesion molecule L1 (CHL1) belongs to the transmembrane adhesion molecule of the immunoglobulin superfamily and exhibits biological functions similar to that of L1. CHL1 contains an N-terminal signal sequence, six immunoglobulin (Ig)-like domains, five fibronectin type III repeat (FN III) sequences, a transmembrane domain and a conserved intracellular domain containing the sequence that functions in the cell skeleton protein anykrin recognition sequence (FIGAY; Holm et al., 1996; Zhang et al., 1998; Maness and Schachner, 2007). CHL1 was identical to melanoma cell adhesion molecule (MCAM), which was previously reported as one of the extravillous trophoblasts (EVT) markers (Higuchi et al., 2003). CHL1 is located within or near the uveal melanoma susceptibility locus UVM2 at 3p25 (Tschentscher et al., 2003). In addition, CHL1 interacts genetically with both CTF7/ECO1 and CTF18/CHL12 to modulate sister-chromatid cohesion (Skibbens, 2004).

The cleavage and release of the CHL1 extracellular domain initiates autocrine signaling and reduces cellular adhesion to promote cell motility (Katic et al., 2014). In the central nervous system, CHL1 promotes Purkinje and granule cell survival and granule cell migration during cerebellar development (Jakovcevski et al., 2009). In *Caenorhabditis elegans*, the chl-1 gene is required for normal development and fertility, whereas CHL1 mutations can lead to lineage-independent cell proliferation defects (Chung et al., 2011). CHL1 dysfunction has been implicated in abnormal thalamocortical circuitry, schizophrenia and autism (Morag et al., 2011). Mutations in the coding region of CHL1 are involved in the etiology of schizophrenia in both Chinese and Japanese populations (Sakurai et al., 2002; Chen et al., 2005). Patients with heterozygous deletion of CHL1 gene can suffer cognitive impairment (Tassano et al., 2014). Research on the roles of CHL1 in tumorigenesis has gradually attracted attention (He et al., 2013). Overexpression of CHL1 was also observed in serous epithelial ovarian cancers (EOCs; Manderson et al., 2009). However, single-nucleotide polymorphism (SNP)-mass array demonstrated the absence and down-regulation of CHL1 expression in primary esophageal squamous cell carcinoma (ESCC) tumors and ESCC cell lines (Qin et al., 2008). Down-regulation/silencing of CHL1 is present in a majority of primary tumors, and its up-regulation is associated with invasive/metastatic growth. In one study, frequent down-regulation of CHL1 was detected in 11 types of cancer, mainly including breast, kidney, colon, thyroid, and stomach. In contrast, only five types (lung, ovary, uterus, liver and trachea) of cancer exhibited frequent up-regulation (Senchenko et al., 2011). These combined data indicated that the functional expression of CHL1 is tumor phenotype dependent. However, CHL1 expression in the development, metastasis, and progression of gliomas both *in vitro* and *in vivo* remains unclear.

To address this issue, we systematically investigated the roles of CHL1 in glioma behaviors mainly using siRNA targeting CHL1 in glioma cells. We evaluated the roles of CHL1 in cell proliferation, metastasis, colony formation, and AKT1 and ERK signaling in these cells. Finally, siRNA targeting CHL1 was intratumorally administered to U-87 MG cell-derived subcutaneous xenografts to further confirm the observations *in vitro*. In summary, CHL1 is vitally involved in the regulation of the occurrence and development of glioma. Targeting the role of CHL1 may represent a promising therapeutic means for the management of glioma.

## Materials and Methods

### Animals

Nude mice were purchased from Beijing Vital River Animal Center (Beijing, China). All the procedures related to handling, care, and treatment in the present research were performed according to the guidelines approved by Institutional Animal Care and Use Committees (IACUC) of Shantou University Medical College.

### Cell Culture and CHL1 siRNA Transfection

Normal human astro glia cell HEB cell line and human glioma cell lines U251 and SHG44 and human glioblastoma U-87 MG cell line (Chinese Type Culture Collection, Shanghai, China) were cultured in Dulbecco’s modified Eagle’s medium (DMEM, Thermo Scientific HyClone, Beijing, China) supplemented with 50 U/ml of a penicillin/streptomycin mixture (Solarbio Biotech Corp. Beijing, China) and 10% fetal bovine serum (Sijiqing Biotech Corp, Hangzhou, China). All cells were routinely grown in 75-cm^2^ cell culture plates (Corning Inc., Corning, NY, USA) at 37°C with 5% CO_2_ in a humidified atmosphere. The cells were collected in logarithmic phase for the following experiments. On the day before transfection, cells were digested by trypsin (0.25%, Solarbio Biotech Corp., Beijing, China), counted and seeded in a six-well plate at an optimal concentration. When the cells achieved 80% confluence, the medium was changed with serum-free DMEM, and cells were incubated overnight. Control siRNA or siRNA targeting CHL1 (10 nM for both; Table 1) complexed with Entranster™-R4000 (Cat. No. 4000-3, Engreen, Beijing, China) was transfected into three cell lines. In the vehicle control group, cells were treated with the same volume of transfection reagent. The efficiency of CHL1 siRNA was confirmed by RT-PCR and Western blot.

**Table 1:** Sequences for random control siRNA and siRNAs against CHL1.

siRNA	Sequence	
	Sense (5′-3′)	Anti-sense (5′-3′)
Random control	UUCUCCGAACGUGUCACGUtt	ACGUGACACGUUCGGAGAAtt
CHL1	GGAGCUAAUUUGACCAUAUtt	AUAUGGUCAAAUUAGCUCCtt

### Cell Viability Assay

U251, SHG44 and U-87 MG cells were seeded onto the 96-well plate at 5000 cells/well in 200 μl of DMEM supplemented with 10% FBS. These cells were transfected with control siRNA or siRNA targeting CHL1 or treated with transfection reagent as mentioned above. Transfected and non-transfected cells were incubated under the conditions of 5% CO_2_, saturated humidity, and 37°C for 24, 48, 72, and 96 h. Then, 20 μl of 5 mg/ml MTT (Beyotime, Jiangsu, China) was added to each well, and cells were further cultured for 4 h. Then, the culture medium was removed, and 150 μl of dimethyl sulfoxide (DMSO, Sigma) was added. The optical density was measured at 570 nm using a multiwell spectrophotometer (Infinite M1000, Tecan, Switzerland). Cell growth curves were plotted using the average absorbance at 570 nm from triplicate samples of three independent experiments.

### Colony Formation Assay

Forty-eight hours after transfection, U251, SHG44 and U-87 MG cells were seeded onto six-well plates at a density of 500 cells/well in triplicate. After 14 days of culture, cells were fixed with methanol, stained with 0.5% crystal violet, and visualized under a phase-contrast light microscope (Olympus, IX51, Japan). Cells were then lysed in 1% SDS, and the colony formation was indexed by the optical density measured at 564 nm using a multiwell spectrophotometer (Infinite M1000, Tecan, Switzerland).

### Cell Senescence Assay

CHL1 siRNA was tested in U251, SHG44 and U-87 MG cells to assess the effect of CHL1 on cell senescence. Cells (1 × 10^5^ cells/well) in culture medium were allowed to adhere overnight to 24-well plates. When 80% confluence was achieved, the medium was aspirated and replaced with fresh medium containing RNA-siRNA-mate complexes (10 nM of either the control or CHL1 siRNA per well). The cells were further cultured for 48 h. Cells treated with the same volume of siRNA-mate were used as the vehicle control. After 48 h of treatment, the cells were fixed. β-galactosidase/X-Gal complex was added to each well and incubated overnight at 37°C according to the manufacturer’s protocol (Beyotime, Jiangsu, China). Cell senescence was indexed by the activation of β-galactosidase reflected by the development of deep blue color of X-Gal (Dimri et al., 1995). Photographs were obtained from at least five random bright-field areas. The percentage of deep blue-stained cells was counted to indicate the senescence response of cells to CHL1 down-regulation.

### Transwell Migration Assay

Given that L1 potentiates the migration of glioma cells, we hypothesized that CHL1 may also possess similar functions. We then tested the potential role of CHL1 for inducing glioma cell migration using the Transwell migration assay (Zhao and Schachner, 2013). For the Transwell migration assay, U-87 MG, SHG44, and U251 cells were pretreated individually with the vehicle control, CHL1 siRNA, and random control siRNA in DMEM for 48 h. The culture medium was then aspirated, and cells were resuspended in DMEM and seeded onto the upper chamber (1 × 10^5^ per well) of each Transwell insert consisting of a filter (Becton Dickinson Labware, Franklin Lakes, NJ, USA) with 8-μm pores. The underside of the filter was pretreated with 100 μg/mL fibronectin in PBS (Millipore) to ensure the attachment of the migrated cells to this side of the filter. The lower chamber was loaded with 500 μl of DMEM only. At 18 h after plating, the cells that had failed to move to the underside of the filter were removed using a cotton-tipped applicator. The cells retained on the underside of the filter were rinsed 3× with PBS and fixed in 4% paraformaldehyde in PBS. The migrated cells were stained with 0.1% crystal violet in acetic acid to determine their morphology. The crystal violet staining was evaluated at 200× magnification with bright field microscopy. The number of cells in each field was counted using Image Tool II software.

### Xenograft Studies

For subcutaneous implantation, 10 4-week-old female BALB/c nude mice (Vital River, Beijing, China) were randomly divided into control siRNA group (*n* = 5) and siRNA targeting CHL1 group (*n* = 5). Mice were anesthetized with 100 mg·kg^−1^ ketamine, and 5 × 10^5^ U-87 MG cells were injected into the right flank near the upper extremity. After 4 weeks, tumor length and width were measured with calipers in cephalad-to-caudad and left-to-right dimensions, and measurements continued at one-day intervals. Tumor volume was calculated each day using the formula: volume = length × width^2^ × 0.5 and expressed in mm^3^. When the tumor volume reached approximately 150 mm^3^, control siRNA or CHL1-siRNA complexed with Entranster™-*in vivo* were intratumorally injected at 2 mg/kg for the 1st time and at 4 mg/kg 7 days after the 1st injection (EngreenBiosystem Co. Ltd., Beijing, China) was undertaken. The second intratumoral injection was performed on the 7th day after the 1st intratumoral injection. After the 16th day of measurements, mice were anesthetized and euthanized by decapitation to remove the tumors.

### RNA Isolation and Reverse Transcriptase PCR (RT-PCR) Analysis

Total RNA from glioma cells was extracted using RNAiso extraction kit (Tiangen, Beijing, China) according to the manufacturer’s protocol, and reverse transcription was performed using StarScrip II First-strand cDNA Synthesis Mix (GenStar, Beijing, China). A 35-cycle PCR using the following conditions was performed (except for the GAPDH primer set): 94°C for 2 min, 94°C for 30 s, 59°C for 20 s, 74°C for 40 s and a final extension step at 72°C for 10 min. For GAPDH cDNA amplification, 28 reaction cycles were employed. We subjected 5 μl of the PCR products to gel electrophoresis using a 2.0% agarose gel (Gene Choice) containing Gelred (1:10,000; Biotium, Hayward, CA, USA). The bands were identified under UV light. The primers used for PCR for detecting the mRNA expression were listed as follows: hCHL1-forward primer: 5′-TCAAAGGAAGCCTTCGGTCC-3′ and hCHL1-reverse primer: 5′-TAGATCCAGCGTAGGCACCA-3′; GAPDH forward primer: 5′-TATAAATTGAGCCCGCAGCC-3′ and GAPDH reverse primer: 5′-TTCCCGTTCTCAGCCTTGAC-3′. The signal intensity was quantified using Image Tool II software via average densitometry multiplied by the number of pixel (National Institutes of Health, Bethesda, MD, USA). The relative mRNA level of a protein was indexed by its signal intensity to that of GAPDH.

### Western Blot Analysis

The cells and tumor samples were lysed in a RIPA buffer mixture (Solarbio Biotech, Beijing, China) supplemented with PMSF (1:200, Solarbio Biotech). The cell lysates were centrifuged at 14,000× *g* for 15 min at 4°C, and the supernatants were collected for Western blot analysis (Zhao W. and Ren, 2011; Zhao W. J. and Ren, 2011). Equivalent quantities of the lysates from the cells were heated at 95°C in 20% sample loading buffer (0.125 M Tris-HCl, pH 6.8, 20% glycerol, 10% SDS, 0.1% bromophenol blue and 5% β-mercaptoethanol), resolved using an 8% SDS-PAGE and electroblotted onto polyvinylidenedifluoride membranes (PVDF, Millipore, Billerica, MA, USA). Non-specific protein binding sites were blocked with 5% BSA diluted in Tris-buffered saline (TBS, pH 7.3) buffer containing 0.05% Tween-20 (TBST). Membranes were incubated with a rat anti-human CHL1 antibody that specifically targets the extracellular domain of CHL1 (1:500, R&D Systems, cat. no. MAB2126, Minneapolis, MN, USA), mouse monoclonal anti-Bcl-2 antibody (1:1000, cat. no. sc-7382, Santa Cruz, CA, USA), rabbit polyclonal anti-Bax antibody (1:1000, cat. no. sc-526, Santa Cruz), rabbit polyclonal anti-PCNA antibody (1:1000, cat. no. sc-7907, Santa Cruz), rabbit polyclonal anti-caspase-3 antibody (1:1000, cat. no. sc-7148, Santa Cruz), mouse monoclonal anti phosphorylated extracellular signal regulated kinase 1/2 (anti-pErk1/2) antibody (1:1000, cat. no. sc-7383, Santa Cruz), mouse monoclonal anti-Erk1/2 antibody (1:1000, cat. no. sc-135900, Santa Cruz), mouse monoclonal anti-pAkt1 antibody (1:1000, cat. no. sc-81433, Santa Cruz), mouse monoclonal anti-Akt1 antibody (1:1000, cat. no. sc-55523, Santa Cruz) and mouse monoclonal anti-glyceraldehyde-3-phosphate dehydrogenase (GADPH) antibody (1:1000, cat. no. sc-365062, Santa Cruz) overnight at 4°C. After washing the membrane with TBST three times at room temperature (5 min each wash), the membranes were further incubated with horseradish peroxidase conjugated goat anti mouse secondary antibody (1:1000, cat. no. BA1051, Boster Biological Technology, Wuhan, China), anti rabbit secondary antibody (1:1000, cat. no. BA1055, Boster Biological Technology), or rabbit anti-rat secondary antibody (1:1000, cat. no. BA1058, Boster Biological Technology) for 1 h. Subsequently, the membranes were washed with TBST three times (5 min each wash) at room temperature. The immunoreactive bands were visualized using an enhanced chemiluminescence kit (Bio Rad Laboratories, Richmond, CA, USA) and an imaging system (Alpha Innotech, San Leandro, CA, USA). The signal intensity was quantified using Image Tool II software via average densitometry multiplied by the number of pixels (National Institutes of Health, Bethesda, MD, USA). The relative expression level of the protein under study was indicated by the ratio of its signal intensity to that of GAPDH.

### Immunohistochemical Analysis

Immunohistochemical staining of paraffin sections was performed as described (Zhao et al., 2009). The human U-87 MG glioma cell xenograft tissues were cryosectioned at 8 μm thickness. Antigen retrieval was performed using 10 mM citrate buffer (pH 6.0), and endogenous peroxidase clearance was performed by incubation in 3% H_2_O_2_. Then, sections were blocked with 10% normal goat serum in PBS at room temperature for 30 min, and samples were subjected to incubation with the following primary antibodies: rat anti-human CHL1 antibody (1:100, cat. no. MAB2126, R&D Systems), rabbit polyclonal anti-PCNA antibody (1:200, cat. no. sc-7907, Santa Cruz), rabbit polyclonal anti-caspase-3 antibody (1:200, cat. no. sc-7148, Santa Cruz), rabbit polyclonal anti-GFAP antibody (1:500, cat. no. BA0056, Boster Biological Technology) at 4°C overnight. Bound antibody was visualized using the AEC method. Counterstaining was performed with Mayer’s hematoxylin. H&E (Zhongshan Goldbridge Biotechnology Co., LTD, Beijing, China) and immunohistochemical stainings were analyzed using a Jiangnan light microscope (DN-10B, Jiangnan, Nanjing, Jiangsu).

## Statistical Analysis

*In vitro* experiments were repeated at least three times using independent culture preparations. All data are presented as group mean values with standard error of the mean (SEM). Statistical analyses were performed using SPSS (Statistical Package for the Social Sciences) 10.0 software (SPSS, Chicago, IL, USA). The data from CHL1 siRNA group were compared with those from either vehicle control or control siRNA group by using Student’s *t*-test for independent samples. *p*-values < 0.05 were considered statistically significant.

## Results

### Comparison of the Protein Levels of CHL1 in 3 Glioma/Glioblastoma Cell Lines with that in Normal Human Glia HEB Cells

**Figure 1:**
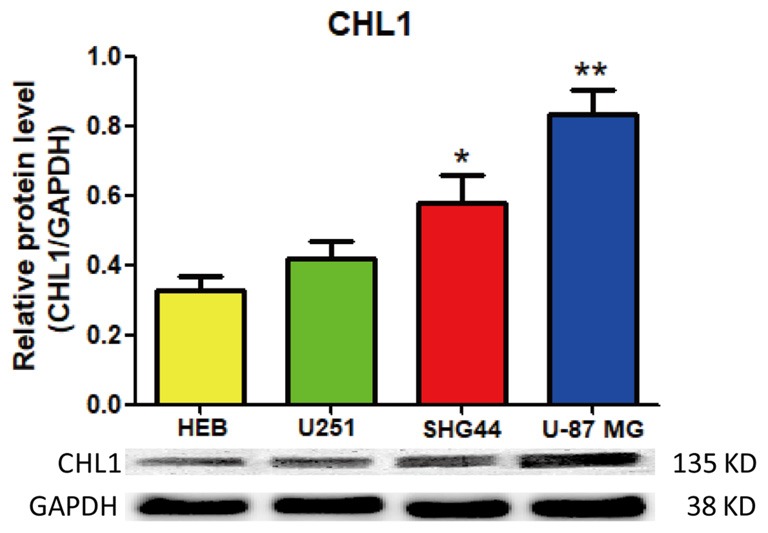
Western blot analysis of the protein levels of CHL1 detected in normal human glial HEB cells and 3 glioma/glioblastoma cell lines. CHL1 was weakly expressed in normal human HEB glial cells. Its levels in all the 3 glioma/glioblastoma cells were higher than that in normal human HEB glial cells, with the statistical significance detected in SHG44 cells (*p < 0.05 vs. HEB cells) and U-87 MG cells (**p < 0.01 vs. HEB cells). *n* = 3 for each group. Student’s *t*-test for independent samples was used.

We first used Western blot to evaluate and compare the expression of CHL1 protein in HEB and three human glioma/glioblastoma cell lines. As demonstrated in Figure 1, CHL1 was weakly expressed in normal human HEB glial cells. Its levels in all the 3 glioma/glioblastoma cells were higher than that in normal human HEB glial cells, with the statistical significance detected in SHG44 cells (*p* < 0.05 vs. HEB cells) and U-87 MG cells (*p* < 0.01 vs. HEB cells). Western blot of CHL1 using samples from all the four cell lines on the uncut PVDF membrane suggests that the CHL1 antibody used in the present study is highly specific (Supplementary Figure S1).

### Down-Regulation of CHL1 Expression Affects Glioma Cell Proliferation and Survival

**Figure 2:**
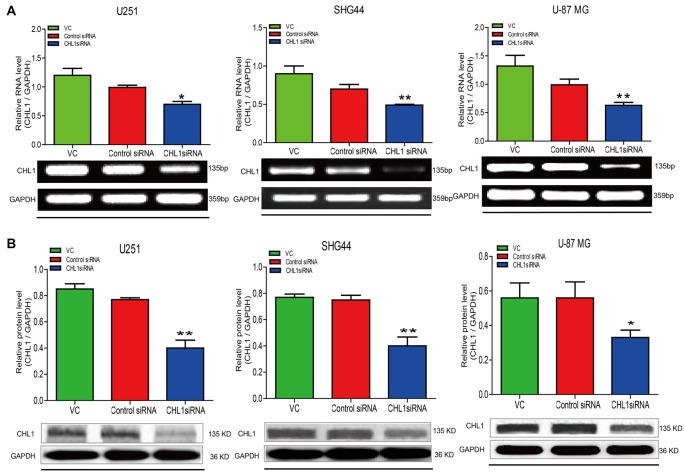
Treatment of siRNA targeting CHL1 in three human glioma cell lines. Total RNA was isolated from U251, SHG44 and U-87 MG cells treated with vehicle control (vc), control siRNA (control siRNA) or siRNA targeting CHL1 (CHL1 siRNA). RT-PCR and Western blot analysis were then used to measure both relative mRNA and protein levels of CHL1. **(A)** RT-PCR analysis of the mRNA levels of CHL1 in U251, SHG44 and U-87 MG cells treated with vehicle control (vc), control siRNA and siRNA targeting CHL1, and **(B)** Western blot analysis of the protein levels of CHL1 detected in U251, SHG44 and U-87 MG cells treated with vehicle control (vc), control siRNA and siRNA targeting CHL1. Data are presented as means ± standard error of the mean (SEM) (*n* = 3, *p < 0.05; **p < 0.01, independent Student’s *t*-test).

**Figure 3:**
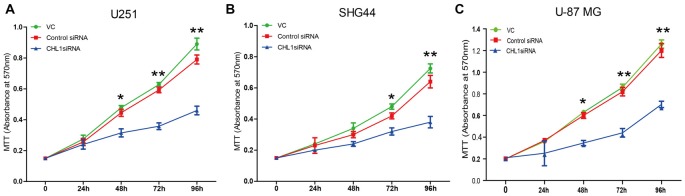
Knockdown of CHL1 affects the proliferation and survival of U251, SHG44 and U-87 MG glioma cells. Cells were seeded on 96-well plates in triplicate, and proliferation rates were measured by MTT assay to evaluate the effect of CHL1 on the proliferation of U251, SHG44 and U-87 MG glioma/glioblastoma cells. **(A–C)** Changes of the proliferation rate in U251** (A)**, SHG44 **(B)**, and U-87 MG **(C)** cells treated with vehicle control (vc), control siRNA (control siRNA) or siRNA targeting CHL1 (CHL1 siRNA). The data were expressed as the means ± SEM of three independent experiments (*p < 0.05 and **p < 0.01 vs. either vehicle control or control siRNA; independent Student’s *t*-test).

We then explored whether glioma cell oncogenicity is dependent upon CHL1. We reduced CHL1 expression using Entranster™-R4000-mediated CHL1-siRNA transfection inU251, SHG44 and U-87 MG glioma cells to specifically knock down endogenous CHL1 expression. CHL1 expression was significantly suppressed by siRNA targeting CHL1 mRNA and protein levels in all three cell lines investigated (Figures 2A,B). As seen from the MTT assay, compared with the vehicle control group and negative control group, knocking down CHL1 significantly reduced the viability of U251 cells at 48, 72 and 96 h (*p* < 0.05 vs. controls at 48 h, and *p* < 0.01 vs. controls at 72 h and 96 h; Figure 3). A similar pattern of cell viability inhibition in response to CHL1 knockdown was observed in both SHG44 glioma and U-87 MG glioblastoma cells, with remarkable viability inhibition observed at both 72 h and 96 h time points (*p* < 0.01 vs. controls at both time points; Figure 3). These results suggested that CHL1 is involved in the viability of glioma/glioblastoma cells.

### Reducing CHL1 Expression Promotes Cell Senescence

**Figure 4:**
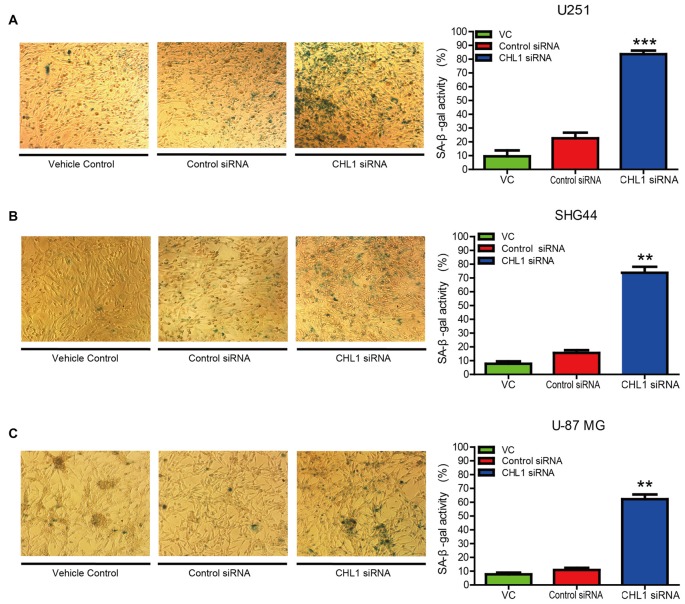
Knockdown of CHL1 affects the senescence of glioma/glioblastoma cells *in vitro*. **(A–C)** U251 **(A)**, SHG44 **(B)** and U-87 MG **(C)** cells were seeded onto 24-well plates and treated with vehicle control, control siRNA and siRNA targeting CHL1, and senescent cells were then detected by senescence-associated β-galactosidase staining (200×). The data were expressed as the means ± SEM from four independent experiments (**p < 0.01; ***p < 0.001 vs. either vehicle control or control siRNA; Independent Student’s *t*-test).

To investigate whether down-regulation of CHL1 leads to cell senescence, we performed senescence staining via a β-galactosidase activity assay. After glioma cells were transfected with CHL1 siRNA or negative control siRNA, cells were fixed and stained for β-galactosidase activity using the X-Gal substrate at 37°C for 24 h. In the vehicle control with U251 cells, 10.16% of the total cells were X-Gal-positive, whereas the average percentage of X-Gal-positive cells upon treatment with negative control siRNA and CHL1 siRNA were 21.67% and 82.46%, respectively. Thus, knocking down CHL1 expression induced a significant increase in SA-β-gal-positive senescent cells compared with the vehicle control and negative control siRNA (*p* < 0.001 for both; Figure 4A). Similarly, SHG44 and U-87 MG cells were also sensitive to CHL1 knockdown-induced senescence. Both the CHL1 knockdown groups (74.89% and 61.27% for SHG44 and U-87 MG cells, respectively; Figures 4B,C) exhibited a remarkable increase in the number of SA-β-gal-positive cells compared with vehicle control (9.87% and 8.36% for SHG44 and U-87 MG cells, respectively; *p* < 0.01 vs. vehicle control for both cell lines; Figures 4B,C) and negative control siRNA (17.56% and 10.53% for SHG44 and U-87 MG cells, respectively; *p* < 0.01 vs. negative control for both cell lines; Figures 4B,C). These results indicated that knocking down CHL1 expression significantly promoted glioma cell senescence.

### Knockdown of CHL1 Expression Inhibits Glioma Cell Colony Formation *in Vitro*

**Figure 5:**
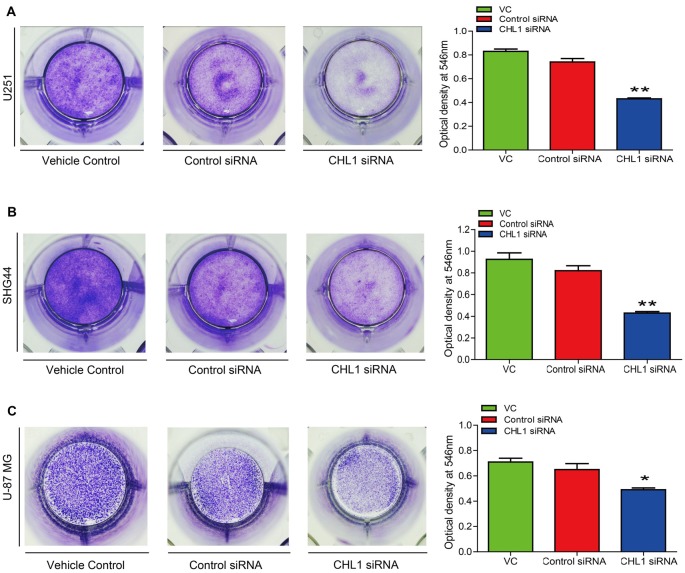
Knockdown of CHL1 reduced colony formation capacity of glioma/glioblastoma cells *in vitro*. Cell colony was stained by crystal violet, which was then dissolved in 1% SDS and the optical density was measured at 546 nm under a microplate reader. **(A)** The colony formation assay revealed that knockdown of CHL1 reduced the colony formation of U251 cells, as was revealed by the optical density detected at 546 nm from three independent experiments (*p < 0.05; **p < 0.01 vs. either vehicle control or control siRNA). **(B,C)** Similar results were found in SHG44 **(B)** and U-87 MG **(C)** cells for the colony formation experiment. The data were expressed as the means ± SEM from 4 independent experiments (*p < 0.05 and **p < 0.01 vs. both vehicle control and control siRNA; independent Student’s *t*-test).

To test whether knocking down CHL1 expression suppresses colony formation, we performed a colony formation assay using U251, SHG44 and U-87 MG cells. After 14 days, the cells were stained with crystal violet and imaged to analyze colony formation rates. Then, crystal violet was dissolved in 1% SDS, and the optical density was measured at 546 nm using a microplate reader. The results demonstrated that CHL1 down-regulation induced a significant reduction in the colony formation rate compared with the negative control siRNA in U251 cell line (*p* < 0.05, Figure 5A). Similarly, SHG44 and U-87 MG cell lines with CHL1 knocked down also exhibited a remarkable decrease in the colony formation compared with the negative control siRNA group (*p* < 0.01, Figures 5B,C).

### Knockdown of Expression Inhibits Cell Invasion *in Vitro*

**Figure 6:**
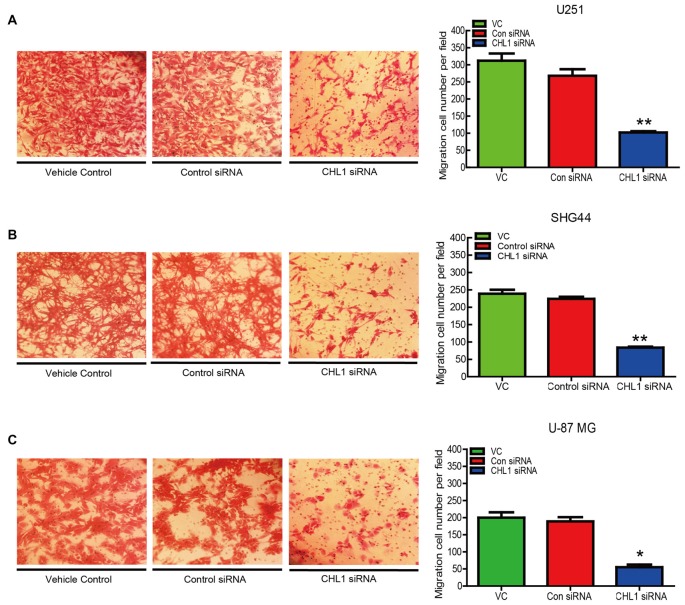
Knockdown of CHL1 suppressed the migration of glioma/glioblastoma cells *in vitro*. Transwell migration assays were carried out using U251 **(A)**, SHG44 **(B)** and U-87 MG **(C)** cells transfected with CHL siRNA. Representative fields containing migrated cells attached to the underside of the membrane were presented. The migration ability was indexed by the relative number of migrated cells from three independent experiments. The data were expressed as the means ± SEM from 3 independent experiments (*p < 0.05; **p < 0.01 vs. both vehicle control and control siRNA (independent Student’s *t*-test).

The number of invading U251 cells in the CHL1-siRNA-transfected group was 100 ± 9, which was significantly lower than that in the groups of cell streated with either the vehicle control or the negative control siRNA (312 ± 11 and 276 ± 8, respectively; *p* < 0.05 for both comparisons (Figure 6A). In addition, the numbers of invading SHG44 and U-87 MG cells in the CHL1-siRNA-transfected groups were also significantly decreased compared with cells treated with vehicle or negative control siRNA (84 ± 6 vs. 239 ± 7 and 224 ± 4 for SHG44 cells and 54 ± 5 vs. 187 ± 6 and 205 ± 3 for U-87 MG cells, respectively; *p* < 0.001 for comparisons in SHG44 cells, and *p* < 0.01 for both comparisons in U-87 MG cells; Figures 6B,C).

### Knockdown of Expression Affects Cell Survival/Apoptosis Signaling Pathways in Glioma Cells

**Figure 7:**
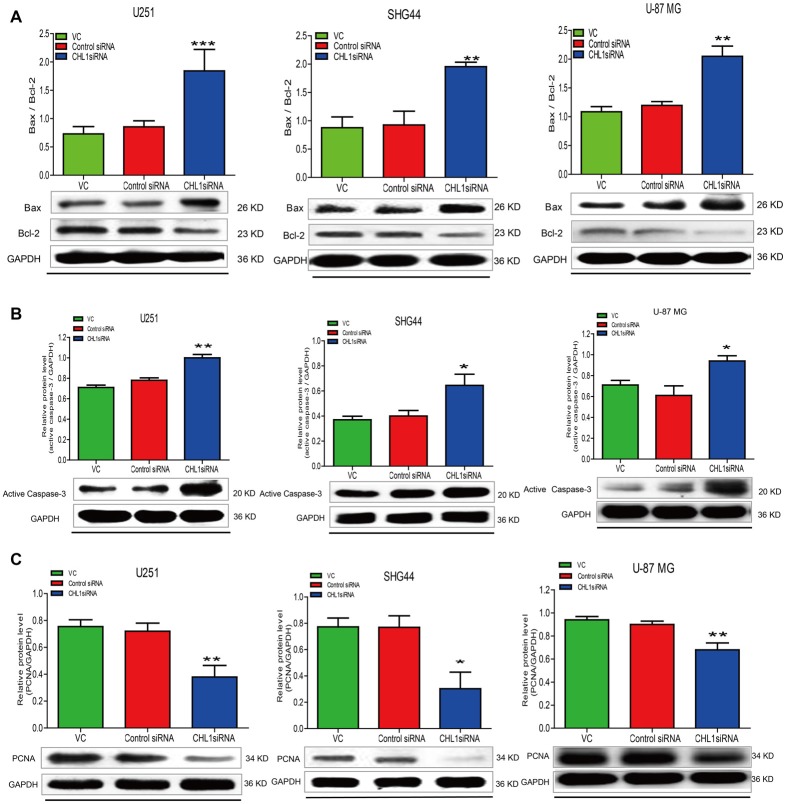
Knockdown of CHL1 affects apoptosis signaling molecules in glioma/glioblastoma cells. U251, SHG44 and U-87 MG cells were seeded onto 48-well plates and treated with vehicle control, control siRNA and siRNA targeting CHL1, respectively. Western blot analysis was performed to determine the levels the apoptosis-related proteins, including changes of the ratio of Bax to Bcl-2 (Bax/Bcl-2) **(A)**, active caspase-3 **(B)** and PCNA **(C)** in glioma/glioblastoma cells. GAPDH was used as the loading control. The data were expressed as the means ± SEM from three independent experiments (*p < 0.05; **p < 0.01; ***p < 0.001 vs. either vehicle control or control siRNA; independent Student’s *t*-test).

To confirm the results of the MTT assay, Western blot was performed to determine the expression of the apoptosis-related proteins Bcl-2, Bax, active caspase-3 and PCNA in the three human glioma cell lines. We found that 48 h post transfection, the ratio of Bax to Bcl-2 was up-regulated in three siRNA-CHL1-transfected cell lines compared with cells treated with vehicle control or the negative control siRNA (*p* < 0.001 for both comparisons in U251 cells, and *p* < 0.01 for both comparisons in regarding SHG44 and U-87 MG cells; Figure 7A). In addition, active caspase-3 protein levels were significantly increased in all 3 siRNA-CHL1-transfected cell lines compared with cells treated with the negative control or vehicle control (*p* < 0.05 for both comparisons in both SHG44 and U-87 MG cells, and *p* < 0.01 for both comparisons in U251 cells; Figure 7B). In addition, PCNA was examined as a possible proliferation indicator in glioma cells. The results demonstrated that PCNA protein levels were significantly reduced in three siRNA-CHL1-transfected cell lines compared with cells treated with vehicle control or negative control siRNA (*p* < 0.05 for both comparisons in SHG44 cells, and *P* < 0.01 for both comparisons in both U251 and U-87 MG cells; Figure 7C). Taken together, these results indicated that CHL1-dependent anti-apoptosis in glioma cells may be partially mediated by regulation of the death receptor signaling pathway composed of Bax, Bcl-2 and active caspase-3.

### Effects of siRNA Targeting CHL1 on Main Signaling Pathways in Human Glioma Cells

**Figure 8:**
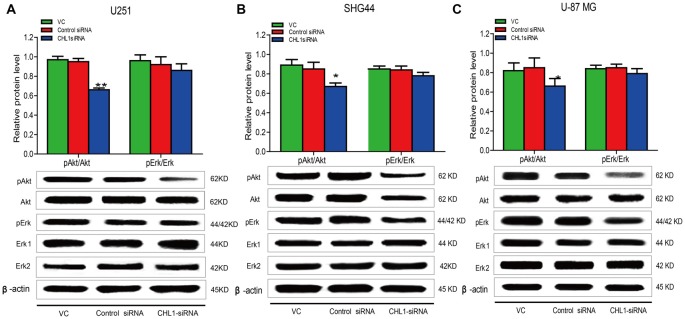
Knockdown of CHL1 reduced the phosphorylation levels of ERK and AKT. Western blot was used to analyze the levels of pAkt and pErk in three cell lines after treatment with vehicle control, negative control and CHL1 siRNA for 48 h. pAkt and pErk protein levels in U251 **(A)**, SHG44 **(B)** and U-87 MG cells** (C)** were presented. GAPDH was used as a loading control. The data were expressed as the means ± SEM from 3 independent experiments (*p < 0.05 and **p < 0.01 vs. both vehicle control and control siRNA; independent Students *t*-test).

Accumulating evidence indicates that the Ras/MAPK/ERK and PI3/AKT signaling pathways contribute to cell growth, proliferation, and survival (Asati et al., 2016). Western blot analysis was performed to determine pErk and pAkt levels in U251, SHG44 and U-87 MG cells in response to CHL1 siRNA treatment for 48 h. We found that 48 h post transfection, pAkt/AKT levels were significantly decreased in three CHL1 siRNA-transfected human glioma cell lines compared with cells transfected with negative control siRNA (*p* < 0.05 for both comparisons in both SHG44 and U-87 MG cells and *p* < 0.01 for both comparisons in U251 cells (Figure 8). However, CHL1 knockdown exhibited no apparent effect on pErk/ERK protein levels at 48 h compared with treatment with either the vehicle control or the negative control siRNA (*p* > 0.05 for both comparisons in all three cell lines).

### CHL1 Regulates U-87 MG Glioma Cell Growth *in Vivo*

**Figure 9:**
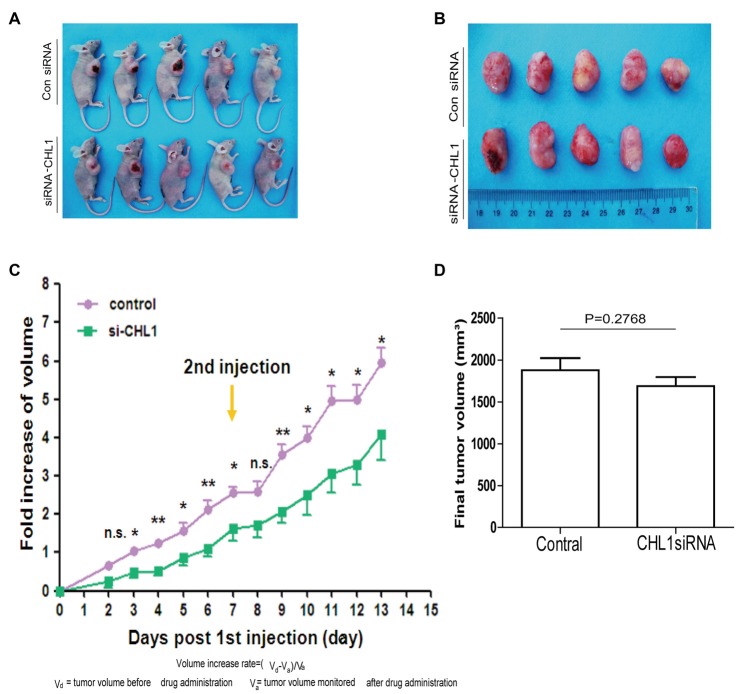
CHL1 regulates growth of U-87 MG glioma cells *in vivo*. **(A,B)** Two weeks after the 1st intratumoral injection, all mice were killed by cervical dislocation. The *in situ* tumors and the dissected tumor tissues were photographed. **(C)** The fold increase of volume at each day points post the 1st intratumoral injection of either control siRNA or CHL1 siRNA complexed with the Entranster™-*in vivo*. **(D)** Column diagram showing the final average tumor volumes from both control siRNA and CHL1 siRNA-treated group (*n* = 5, *p* = 0.2768 vs. the control siRNA group) (*p < 0.05; **p < 0.01 vs. control siRNA; Independent Student’s *t*-test).

We established subcutaneous xenografts of U-87 MG glioblastoma cells in nude mice. To determine whether the tumor formation of glioblastoma oncogenicity is dependent upon CHL1, we reduced CHL1 expression using CHL1-siRNA combined with Entranster™-*in vivo*, which was intratumorally injected to specifically knockdown endogenous CHL1 expression *in vivo*. Intratumoral injection of siRNA targeting CHL1 significantly reduces the fold increases in tumor volume at most time points post siRNA transfection (*p* < 0.05 vs. control siRNA at 3, 5, 7, 10, 11, 12 and 13 day points post 1st siRNA injection; *p* < 0.01 vs. control siRNA at 4, 6, and 9 day points post the 1st siRNA injection; Figures 9A–C). The final tumor volume in mice treated with siRNA targeting CHL1 was lower than that in mice treated with control siRNA, although no statistical significance was detected (1689.41 ± 239.46 vs. 1877.50 ± 325.11 mm^3^, *p* = 0.2768 vs. control siRNA, Figure 9D). However, the total increased tumor volume in mice treated with siRNA targeting CHL1 was significantly lower than that in mice treated with control siRNA (*p* < 0.05 vs. control siRNA, data not shown). These results demonstrate that reducing CHL1 expression can partially inhibit the growth of glioma *in vivo*.

### Morphological Changes in Subcutaneous Xenografts of U-87 MG Cells in Response to CHL1 Transfection

**Figure 10:**
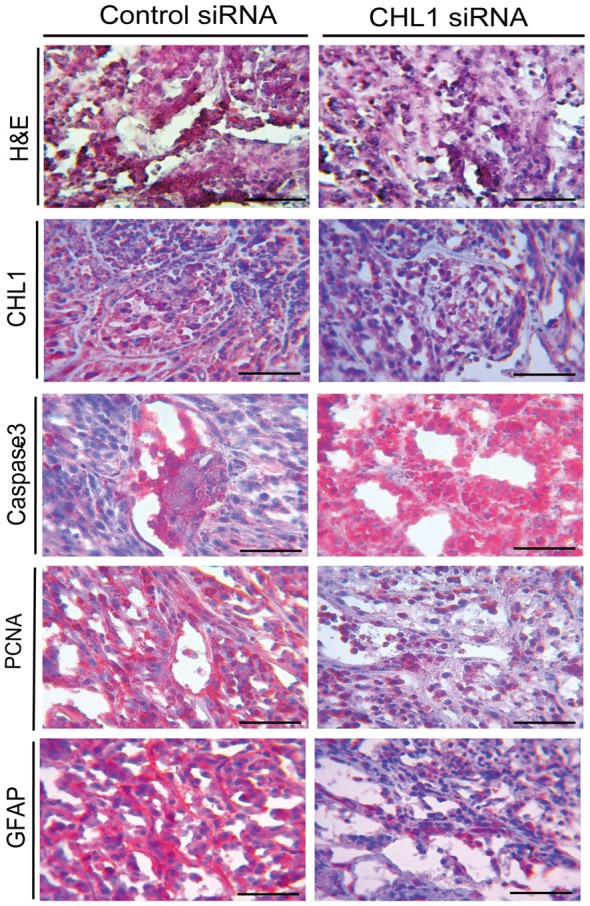
H&E staining and immunohistochemical staining analyses for the CHL1, caspase-3, PCNA and GFAP molecules in glioblastoma xenograft tissues from both control siRNA and CHL1 siRNA-treated groups. Scale bars represent 25 μm.

H&E staining and immunohistochemical staining were performed to analyze changes in the xenograft tumor in response to CHL1 down-regulation. The results demonstrated that the staining intensities of CHL1, PCNA and GFAP were apparently reduced, whereas active caspase-3 intensity was apparently increased (Figure 10).

## Discussion

Despite recent progress in the treatment of melanoma and other tumors, treatment of glioma remains disappointing. Traditional therapeutic means, including radiotherapy and chemotherapy, and neurosurgical removal do not completely eradicate the tumor, and recurrence often occurs (Sathornsumetee et al., 2007). L1 is among the most intensively studied cell adhesion molecules in glioma investigation. Inhibition of L1 expression by siRNA and administration of L1 ectodomain-binding antibodies reduced the migration of glioblastoma cells *in vitro* (Izumoto et al., 1996) and disrupted glioma stem cell proliferation, leading to apoptosis (Bao et al., 2008). Reducing L1 expression *in vivo* also suppressed tumor growth and increased the survival of tumor-bearing animals (Cheng et al., 2011).

The overexpression of cell adhesion molecules, including L1, and specific inhibition of the function of these molecules suggests target-directed and promising therapeutic methods for glioma (Skibbens, 2004; Wolterink et al., 2010). The mAbs specifically reacting with L1CAM efficiently prolong survival and reduce tumor burden in a model of SKOV3ip cells in CD1 nude mice. Antibody-based functional impairment of L1 also altered the expression of cellular genes associated with apoptosis and tumor growth (Wolterink et al., 2010). Additional reports demonstrated that cell adhesion molecule VCAM-1-positive glioblastoma tumor stem cells (GTSC) possess a high rate of proliferation as measured by PCNA expression (Zarnescu et al., 2011). However, neural cell adhesion molecule (NCAM) levels were down-regulated as the malignancy of astrocytomas increased, and this effect is inversely correlated with PCNA (Huang et al., 2001). These data suggested that different cell adhesion molecule members may function differentially in the development of glioma and promoted us to explore the potential roles of CHL1 in promoting glioma cell migration, proliferation and metastasis.

Similar to L1, CHL1 also plays major roles in axonal guidance in the developing brain and mediates the maintenance and remodeling of neural circuits in the adult brain (Sakurai et al., 2002; Hitt et al., 2012). In the present work, CHL1 was lowly expressed in human astroglia cells, but was highly expressed in human glioma cell lines, suggesting its potential roles in the development of glioma. To date, paradoxical data about the roles of CHL1 under the tumorigenic conditions were reported in non-neuronal cell-derived tumors. He et al. (2013) reported that CHL1 is down-regulated in human breast cancer and is related to lower grade. Down-regulating CHL1 expression results in increased proliferation and invasion, and CHL1 deficiency also promotes tumor formation *in vivo*. Knocking down CHL1 expression by miR-10a increased colony formation activity, migration and invasion of human cervical cancer cells, whereas over-expression of CHL1 abolished the effects of miR-10a (Long et al., 2012). In addition, miR-182 promotes cell proliferation and invasion through direct suppression of CHL1 in papillary thyroid carcinoma (PTC; Zhu et al., 2014). miR-590-5p is up-regulated in human cervical cancer and promotes cervical cancer cell growth, cell invasion and colony formation by negatively regulating CHL1 at the posttranscriptional level (Chu et al., 2014). In addition, CHL1 negatively modulates the proliferation and neuronal differentiation of neural progenitor cells (NPCs) by CHL1/ERK1/2 MAPK signaling (Huang et al., 2011). Although CHL1 functions as a putative tumor suppressor during primary tumor growth and is silenced to facilitate *in situ* tumor growth, re-expression of CHL1 on the edge of the tumor mass may promote local invasive growth and enable further metastatic spread in ovary, colon and breast cancers (Senchenko et al., 2011). In addition, CHL1 facilitates the identification of two major histological types of renal cancer as a potentially novel specific biomarker in early pathogenesis (Senchenko et al., 2011). In pituitary adenoma (PA), differential expression of CHL1 may potentially predict a recurrence phenotype (Marko et al., 2012). Senescence is a stress response that stably blocks proliferation and functions as a tumor suppressor in aging and precancerous cells (Collado and Serrano, 2010; Wagner et al., 2015). The lysosomal-β-galactosidase gene is the source of senescence associated-β-galactosidase activity, and that level of lysosomal-β-galactosidase protein increase during senescence (Lee et al., 2006). The presence of senescence in response to CHL1 knockdown indicates the potential role of CHL1 in promoting cell survival during the development of glioma.

The discrepancy of our findings with those observed in non-glia-derived tumors may be due to the fact that glioma cells express a variety of molecules that interact with CHL1. CHL1 also potentiates integrin-dependent haptotactic cell migration toward the extracellular matrix via a potential integrin interaction motif Asp-Gly-Glu-Ala (DGEA) in the sixth immunoglobulin domain (Buhusi et al., 2003). CHL1–CHL1 homophilic interactions inhibit the neurite outgrowth-promoting functions of CHL1, whereas integrins in cis- and trans-configurations are conducive to heterophilic CHL1 interactions (Buhusi et al., 2003; Demyanenko et al., 2004; Jakovcevski et al., 2007, 2009). Observations indicate that homophilic CHL1 trans-interactions regulate differentiation of neuronal progenitor cells at early postnatal stages, whereas heterophilic trans-interactions of CHL1 with vitronectin, integrins and the plasminogen activator system regulate neuritogenesis and neuronal cell migration at a later stage of cerebellar morphogenesis (Hillenbrand et al., 1999; Katic et al., 2014).

The activation of the AKT and ERK signaling pathways plays an important role in the regulation of cell apoptosis, invasion and metastasis of gliomas. Feng et al. (2010) reported that TAM was present in the rat C6 glioma cell line through activation of PI3K/Akt, JNK and ERK signaling pathways to mediate the physiological processes of glioma cell apoptosis. Studies by Li et al. (2013) reported that a DC electric field can activate the AKT and ERK signaling pathways in U251 glioma cells, thus affecting the direction of tumor migration. Wu et al. (2010) found that PI3K-dependent activation of Erk1/2 signaling pathway up-regulates CHL1 expression in the primary culture of astrocytes. Whether the effect of CHL1 on glioma is closely related to the ERK and AKT signaling pathways has not been reported. We demonstrate that downregulation of CHL1 reduces the activation of Akt1 with no apparent effect on Erk1/2 activation. This finding suggests that the modulating role of CHL1 on glioma behaviors may be partially mediated by Akt1 signaling.

Intratumoral injection represents a promising therapeutic method for tumor management via directly introducing siRNA or other forms of RNA into the tumor tissue. Unlike traditional xenografts of cells with a targeting molecule knocked down, *in vivo* knockdown is more similar to the clinical setting. Zheng et al. (2015) introduced shRNA against LunX into lung cancer tissue *in vivo*, which successfully disrupted local invasion, micrometastasis formation and metastatic colonization of lung cancer cells, thus inhibiting the initial and final steps of the invasion-metastasis cascade. Using an *in vivo* tumorigenicity assay, Wang et al. (2012) reported that daily intratumoral injection of let-7a-mimics in nude mice suppresses NIRF expression and reduces tumor growth. Although intratumoral injection of siRNA targeting CHL1 in the present study reduces the volume increase of glioma xenografts, the growth inhibition efficacy is limited. This effect may be partially attributed to low siRNA transfection and expansion due to the high intensity of cell proliferation in the glioma tissue, which is different from that of lung cancer. Increases in the siRNA transfection efficiency may lead to a more promising therapeutic result.

In summary, our work preliminarily demonstrated the roles of CHL1 in the development of gliomas, which may provide a scientific basis for the molecular targeting treatment of gliomas.

## Author Contributions

ZY and QX performed most of the experiments, analyzed the data, and drafted the sections of Materials and Methods, and Results. C-LH was responsible for figure processing and adjusting the format of the draft. QJ and H-FS assisted the performance of some experiments. MS provided suggestions and comments for the project and contributed to writing of the manuscript. W-JZ conceived and designed the experiments, undertook some of the experiments, wrote the manuscript and revised the article critically and approved the final version to be submitted.

## Conflict of Interest Statement

The authors declare that the research was conducted in the absence of any commercial or financial relationships that could be construed as a potential conflict of interest.

## Supplementary Material

FIGURE S1 Specificity testing for CHL1 antibody on an uncut PVDF membrane.
