# Supplementary figures and images for: CHL1 Is Expressed and Functions as a Malignancy Promoter in Glioma Cells
Source: Front Mol Neurosci. 2017 Oct 17;10:324. doi: 10.3389/fnmol.2017.00324 (PMC5650976; doi:10.3389/fnmol.2017.00324)

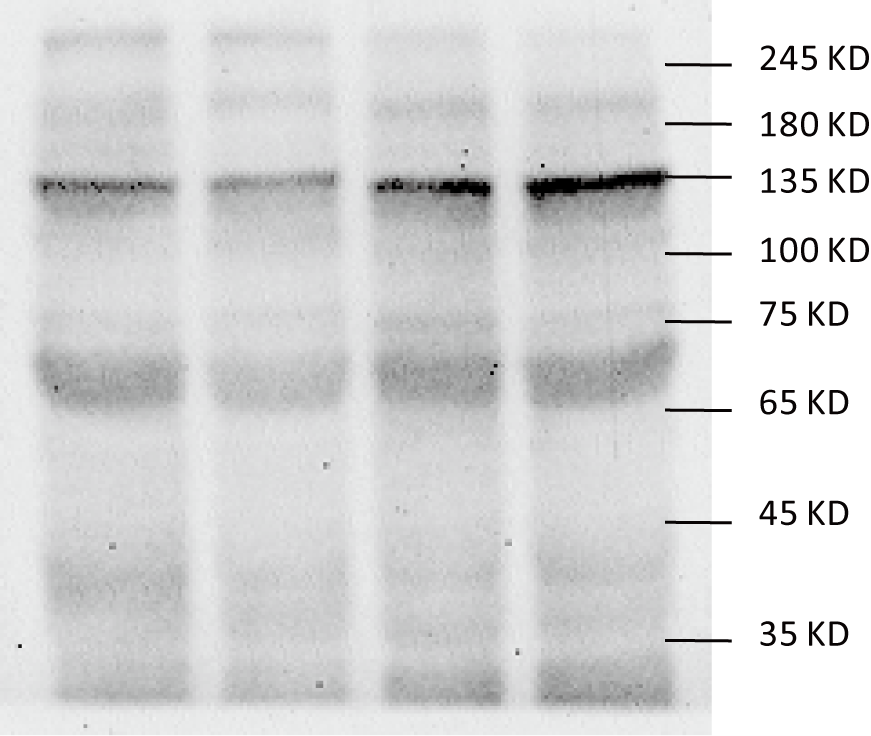

Supplement: FIGURE S1 — Specificity testing for CHL1 antibody on an uncut PVDF membrane. [file Image_1.tif]
